# Supplementary material for: Spatial transcriptomic profiling of coronary endothelial cells in SARS-CoV-2 myocarditis
Source: Front Med (Lausanne). 2023 Mar 9;10:1118024. doi: 10.3389/fmed.2023.1118024 (PMC10034160; doi:10.3389/fmed.2023.1118024)
Supplement: Supplementary file 2 [file Image_1.pdf]

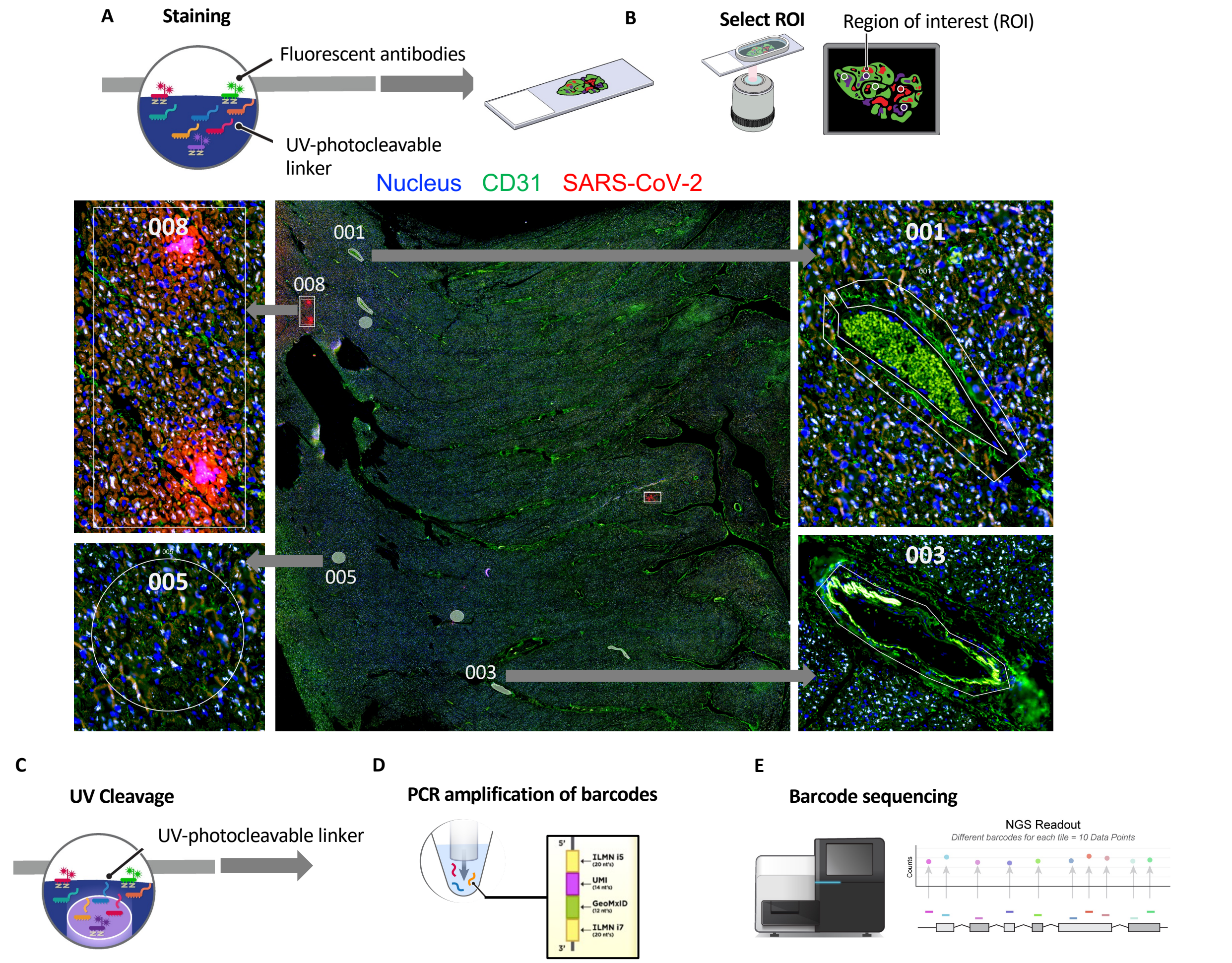

Figure S1. GeoMx Nanostring workflow. Myocardial autopsy samples were processed and stained using standard immunohistochemical protocol with both fluorescent labeled morphology markers and sequence specific RNA probes (A). Morphology markers (CD31 and SARS-CoV-2 nucleocapsid) were used to identify coronary endothelium and areas of SARS-CoV-2 expression as Regions of Interest (ROI). A representative myocardial section with selected ROIs is illustrated (B). After ROI selection, UV light is systematically shone on ROIs to photocleave and decouple barcodes from the RNA probes only from the selected area of interest (C). RNA probes for the ROI were aspirated and deposited into unique wells of a 96 well plate (D). Five probes per mRNA target were used and were coupled to unique molecular barcodes to identify specific gene targets. The Unique Molecular Identifier (UMI) identifies a specific molecule and accounts for amplification bias from PCR. The PCR step adds dual indexing barcodes to identify ROIs and adds Illumina flow cell adapter regions to enable sequencing. The barcodes are sequenced and prepared for further analysis (E).
