# Supplementary figures and images for: Spatial transcriptomic profiling of coronary endothelial cells in SARS-CoV-2 myocarditis
Source: Front Med (Lausanne). 2023 Mar 9;10:1118024. doi: 10.3389/fmed.2023.1118024 (PMC10034160; doi:10.3389/fmed.2023.1118024)

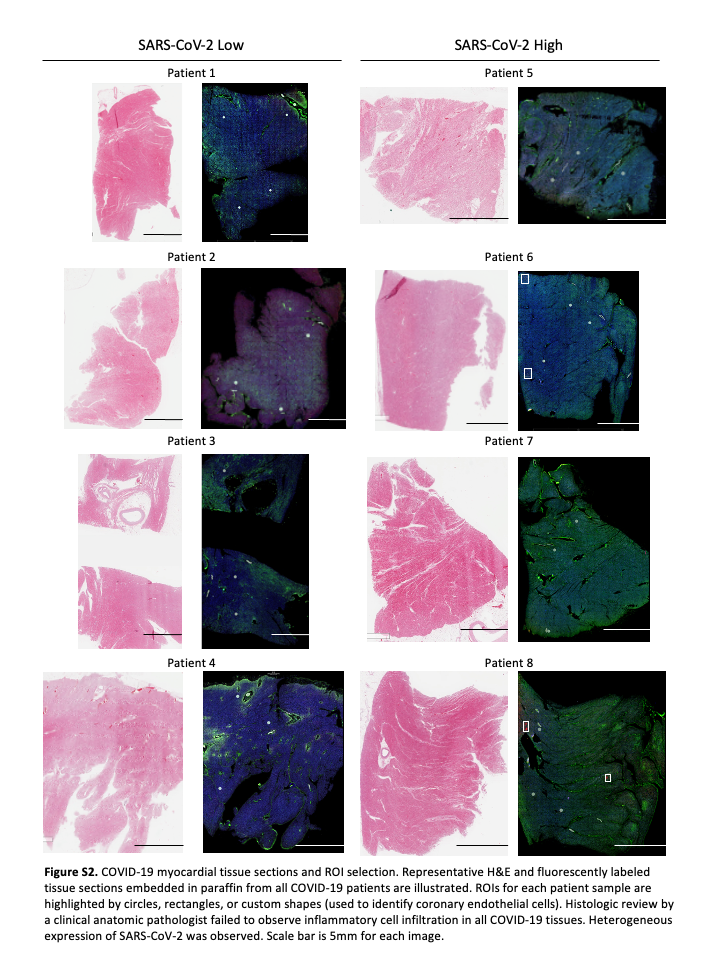

Supplement: Supplementary file 3 [file Image_2.tiff]
