# Supplementary material for: Spatial transcriptomic profiling of coronary endothelial cells in SARS-CoV-2 myocarditis
Source: Front Med (Lausanne). 2023 Mar 9;10:1118024. doi: 10.3389/fmed.2023.1118024 (PMC10034160; doi:10.3389/fmed.2023.1118024)
Supplement: Supplementary file 4 [file Image_3.pdf]

MYOCARDIAL ROIs

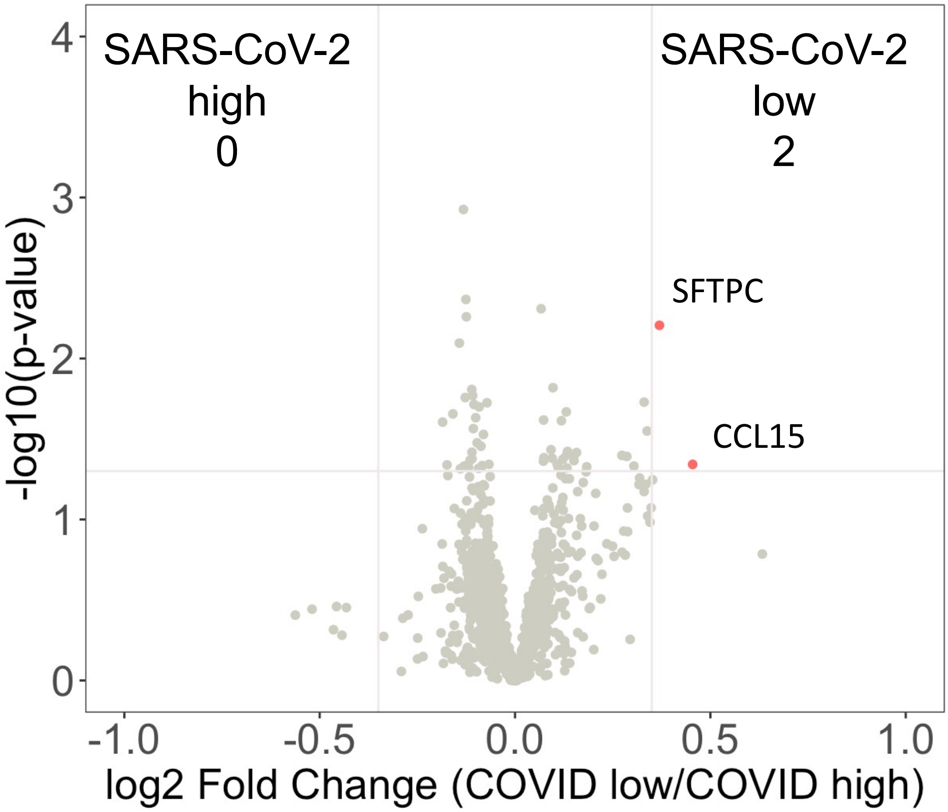

Figure S3. Intensity of SARS-CoV-2 expression does not alter global myocardial expression in severe COVID-19 infection. COVID-19 patient samples were segregated based upon relative expression of SARS-CoV-2 nucleocapsid. Specifically, myocardial specimens were stained for SARS-CoV-2 nucleocapsid and stratified into quartiles based upon relative MFI with the bottom quartile defined as SARS-CoV-2 “Low” and the top quartile defined as SARS-CoV-2 “high”. 1302 genes were above the limit of quantification. Minimal differences in transcriptional programming were observed between SARS-CoV-2 high and SARS-CoV-2 low patient samples. Differential gene expression was defined as p = 0.02 and log2 fold change of 0.4. SARS-CoV-2 high (n = 4 patients, 12 ROIs) and SARS-CoV-2 low (n = 4 patients, 12 ROIs).
