# Supplementary material for: Spatial transcriptomic profiling of coronary endothelial cells in SARS-CoV-2 myocarditis
Source: Front Med (Lausanne). 2023 Mar 9;10:1118024. doi: 10.3389/fmed.2023.1118024 (PMC10034160; doi:10.3389/fmed.2023.1118024)
Supplement: Supplementary file 5 [file Image_4.pdf]

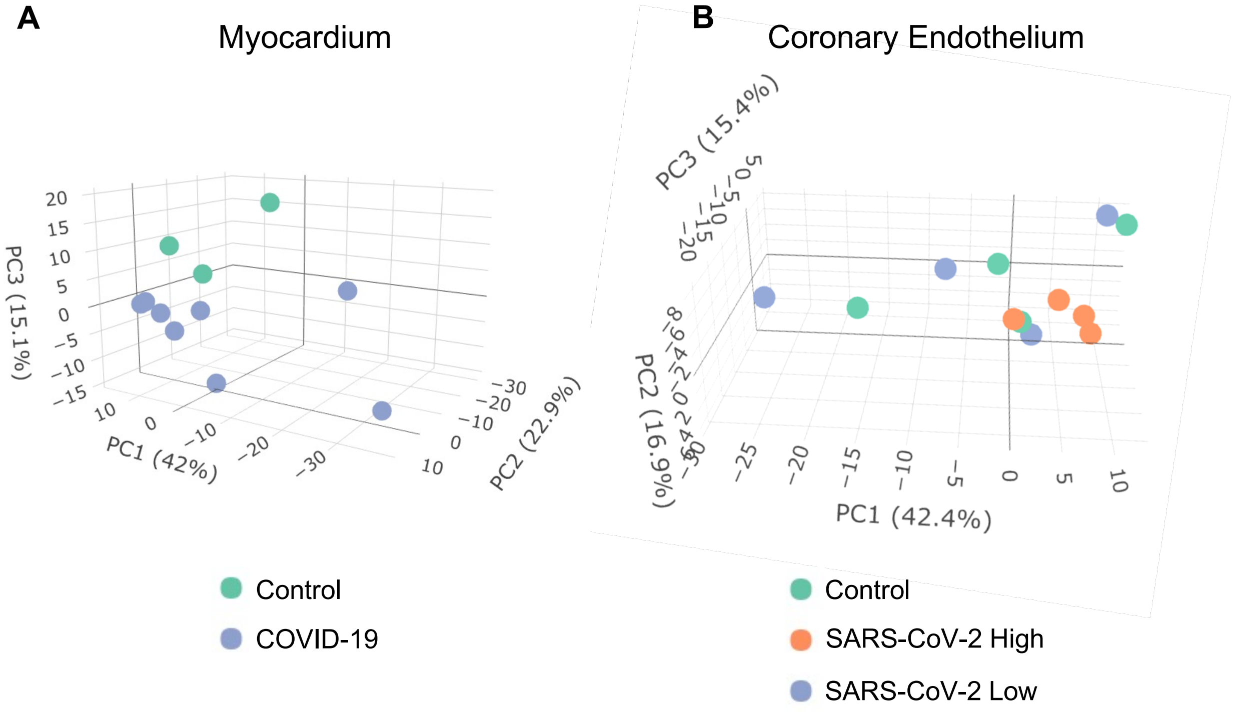

Figure S4. Per patient analysis of spatial transcriptomic data does not influence pattern clustering by Principal Component Analysis (PCA). PCA of unsupervised data from myocardial (A) and endothelial (B) samples were analyzed per patient (as opposed to per ROI) as previously described. Importantly, our original findings were unchanged as myocardial samples continued to show pattern clustering, while no association between SARS-CoV-2 and the endothelium was detected. 1454 and 1302 genes were above the limit of quantification, respectively.
